# The Impact of Mild Chronic Stress and Maternal Experience in the *Fmr1* Mouse Model of Fragile X Syndrome

**DOI:** 10.3390/ijms241411398

**Published:** 2023-07-13

**Authors:** Enejda Subashi, Valerie Lemaire, Valeria Petroni, Susanna Pietropaolo

**Affiliations:** University Bordeaux, CNRS, EPHE, INCIA, UMR 5287, F-33000 Bordeaux, France

**Keywords:** neurodevelopmental disorders, unpredictable stress, ultrasonic communication, Fmr1, maternal care

## Abstract

Fragile X syndrome (FXS) is a pervasive developmental disorder and the most common monogenic cause of autism spectrum disorder (ASD). Female heterozygous (HET) carriers play a major role in the transmission of the pathology and present several FXS- and ASD-like behavioral alterations. Despite their clear genetic origins, FXS symptoms are known to be modulated by environmental factors, e.g., exposure to chronic stress, especially during critical life periods, such as pregnancy. Pregnancy, together with pups’ care, constitutes maternal experience, i.e., another powerful environmental factor affecting several neurobehavioral functions in females. Here we investigated the impact of maternal experience on the long-term effects of stress in *Fmr1*-HET female mice. Our findings demonstrated that the behavioral abnormalities of HET females, i.e., hyperactivity and memory deficits, were unaffected by stress or maternal experience. In contrast, stress, independently of maternal experience, induced the appearance of cognitive deficits in WT mice. Maternal experience increased anxiety levels in all mice and enhanced their corticosterone levels, concomitantly promoting the effects of stress on social communication and adrenal glands. In translational terms, these results advance our understanding of the environmental modulation of the behavioral alterations observed in FXS female carriers and highlight the long-term impact of maternal experience and its interactions with chronic stress.

## 1. Introduction

Fragile X syndrome (FXS) is a major neurodevelopmental disorder (NDD) caused by the absence of fragile X mental retardation protein (FMRP), coded by the *FMR1* X-linked gene and involved in synaptic functionality [1]. FXS patients are characterized by a variety of behavioral symptoms, including anxiety, hyperactivity, cognitive and social deficits [2]. These also include autistic-like symptoms, e.g., reduced social interest and altered communication; FXS indeed represents the most common monogenic cause of autism spectrum disorder (ASD) [3,4,5,6,7]. These FXS- and ASD-like behavioral phenotypes have been successfully recapitulated by the *Fmr1*-KO mouse model that has been extensively employed to investigate the etiopathology and pharmacology of these NDDs. However, the great majority of the existing studies on *Fmr1* mutant mice have focused on males, because of the higher prevalence of FXS and the more marked severity of its symptoms in boys [8,9].

Nonetheless, FXS females are of great interest for human research as they also show several behavioral alterations, including hyperactivity [10], mild cognitive impairments [11,12] and autistic symptoms [5]. Furthermore, FXS women, in particular heterozygous female carriers, play a central role in the progression of this pathology since they are responsible for the affected offspring [13], as affected males are incapable to pass the full mutation to their daughters [14]. Moreover, heterozygous females constitute the majority of FXS women since homozygous *FMR1* mutations are rarely found in the human population [15]. The behavioral investigation of heterozygous females in pre-clinical studies has, therefore, a high translational value; several studies have described the presence of FXS- and ASD-like behavioral abnormalities in *Fmr1* mutant females [16,17,18,19,20,21,22]. These include hyperactivity, anxiety, deficits in spatial memory, alterations in social behaviors and ultrasonic communication [16,17,18,19,20,21,22,23]; these behavioral alterations started to emerge in *Fmr1* heterozygous females during the juvenile age and were mostly confirmed at adulthood [21,22].

Despite their clear and well-defined genetic origins, the pathological phenotypes of FXS and ASD can be strongly modulated by environmental factors, such as exposure to stimulating environments and social interactions [24,25,26], or instead to aversive events [27,28]. Cognitive stimulation and behavioral therapies are known to be able to induce beneficial effects in FXS and ASD patients (e.g., [24,25,26,29]), as confirmed by pre-clinical studies demonstrating that environmental enrichment can rescue several neurobehavioral FXS- and ASD-like alterations in *Fmr1*-KO mice [30,31]. Chronic exposure to stress is instead known to exacerbate the behavioral symptoms of FXS patients [27,28], while the prenatal experience of stressful life events [32,33,34,35] has been shown to be associated with a more elevated risk for ASD in humans [33,36]. Similarly, exposure to chronic stress has been shown to affect the behavioral phenotype of *Fmr1*-KO mice [37,38] and to anticipate its appearance in male and female mutants [22]. Overall, the reactivity to stress of *Fmr1*-KO mice is altered compared to their WT littermates, in line with human data from FXS patients, suggesting a role of gene-environment interactions in the etiopathology of FXS. Nonetheless, the early timing of stress exposure seems to be crucial in determining its behavioral effects in *Fmr1*-KO mice. Pre-natal stressful experiences [22] induced a more marked impact than post-natal stress [37,38] with more pronounced and varied behavioral effects. Nonetheless, these studies have analyzed the effects of gestational stress exclusively on the behavioral phenotypes of the *Fmr1* offspring; therefore, no data are available on the effects of stress during pregnancy on the FXS-like phenotype of *Fmr1* heterozygous dams. Hence, pre-clinical studies on the interactions between maternal experience and stress exposure are needed; they would have a high translational relevance since they would be instrumental in understanding the environmental modulation of the pathological phenotypes induced by the *Fmr1* mutation in FXS female carriers.

The gestational phase represents a critical period, marked by physiological and neurobehavioral changes in the future mother, underpinned by neuroendocrine modifications which have the role of ensuring the proper development of the offspring during embryonic and post-natal life. These changes persist during the early post-partum phases, which together with pregnancy constitute maternal experience; these modifications also involve the activity of the hypothalamic-pituitary-adrenal axis (HPA) [39,40]. Hence, maternal experience may markedly affect the female’s vulnerability to stress. Indeed, it has been shown that exposure to a deleterious/stressful environment during the gestational period may promote the appearance of behavioral disorders in the mother, the risk being greater if associated with a genetic predisposition [40].

Although maternal experience may modulate the vulnerability to stress, for example by magnifying its neurobehavioral and endocrine effects, it is also possible that stress may alter the effects of maternal experience itself. Several behavioral differences, e.g., in anxiety and cognition, have been described between primiparous and nulliparous rodents (especially rats; reviewed in [39,41]). An improvement in spatial memory has been described in rats with maternal experience [42], probably to allow mothers to better care for their pups by reducing the time to find their nests [41]. Interestingly, this cognitive improvement of primiparous versus nulliparous females disappeared following exposure to gestational stress, thus suggesting that stress during pregnancy may eliminate the beneficial behavioral effects of motherhood [43,44]. This hypothesis is further supported by the marked effects of gestational stress on maternal care since previous studies have demonstrated that stressed dams performed less pup-oriented behaviors (e.g., licking/grooming, nursing postures) than non-stressed ones [45,46,47]. It is therefore possible that these effects of stress on maternal care may, in turn, have an impact on other behaviors of the mothers and mediate the stress effects on maternal experience.

Since most of the studies investigating the behavioral effects of maternal experience and its interactions with stress have been conducted in rats, little is known about this issue in the mouse species, which is essential to the study of most genetic models of human diseases. Hence, the impact of stress × maternal experience interactions on the genetic predisposition to develop NDDs has not been studied yet in mice. In the present study, we, therefore, investigated the relative impact of stress and maternal experience on the FXS- and ASD-like behavioral and endocrine phenotypes of the *Fmr1* mutant female mice, following the experimental design illustrated in the method section. *Fmr1*-heterozygous (HET) and wild-type (WT) females, either pregnant or not (primiparous or nulliparous groups), were exposed to one week of unpredictable chronic stress, based on a protocol used in several previous studies on prenatal stress, e.g., [22,48,49,50,51], or left undisturbed (stressed or no-stressed groups). The behavior of all females was evaluated 5 weeks after the end of the differential stress exposure through tests tailoring anxiety and exploration (elevated plus maze and open field), spatial memory (Y-maze), social interaction and ultrasonic communication, i.e., all tests used in previous studies on *Fmr1* females [21,22]. Furthermore, the corticosterone blood levels and adrenal gland weights were assessed one week after the end of behavioral testing. In order to assess the potential role of changes in maternal care in mediating the effects of gestational stress, the maternal behavior of primiparous dams was also assessed during the first week after parturition. Our overall hypothesis was that *Fmr1*-HET females would be more sensitive to the behavioral and endocrine effects of stress and that this sensitivity would be further accentuated by pregnancy/maternal experience. 

## 2. Results

### 2.1. Maternal Behavior

**Figure 1:**
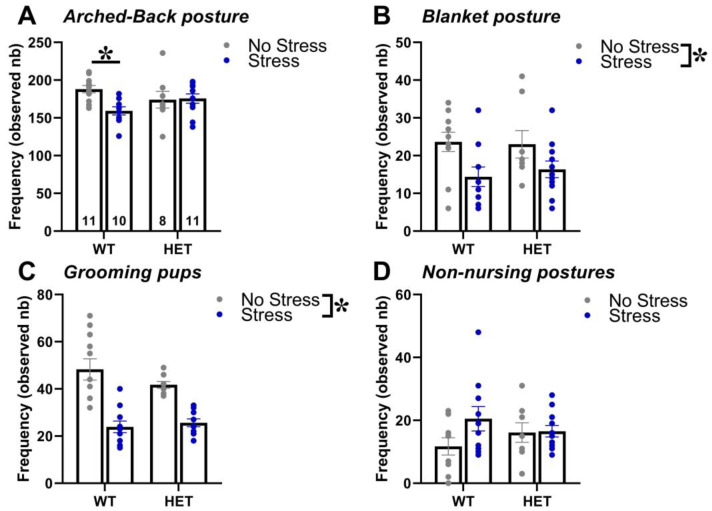
**Maternal behaviors in primiparous dams.** The maternal behavior of all females of the primiparous group (n = 8–11 for each genotype × stress condition, as detailed in (**A**)) was observed in their home cages twice a day for one hour (at 9.00 A.M. and at 5.00 P.M.) from post-natal day (PND) 1 to PND 6, using an instantaneous sampling method (one sampling/2 min). The following items were scored as absolute frequencies by an observer who was blind to the experimental conditions of the breeders [31,52,53]: (i) nursing postures, including arched-back nursing (the female is in an arched position over the nursing pups, (**A**)) and blanket (the female is lying flat on top of the pups, (**B**)), (ii) licking/grooming of the pups (**C**), (iii) non-nursing postures (the female is in contact with the pups, but not nursing, i.e., with no access to the nipples, (**D**)). All behaviors were summed up across the 6 days of scoring. Data are expressed as mean ± standard error of the mean (SEM). * *p* < 0.05.

Stress during pregnancy reduced maternal care and this effect was slightly more pronounced in WT than in HET dams (Figure 1). Indeed, WT stressed dams performed less arched-back postures than controls, an effect that was absent in HET females [interaction genotype × stress: F(1,36) = 4.99, *p* = 0.03, post hoc: WT stress versus WT no-stress; Figure 1A]. Stress decreased the expression of another nursing posture, i.e., blanket, as well as of licking/grooming of the pups, but this effect was observed in both WT and HET dams [stress effect, respectively: F(1,36) = 8.61 and 45.12, *p* < 0.01 and *p* < 0.0001; Figure 1B,C]. No statistically significant effect was detected on non-nursing postures [genotype effect, stress effect and their interaction: F(1,36) = 0.01, 2.45 and 2.02; ns; Figure 1D].

### 2.2. Elevated Plus Maze

**Figure 2:**
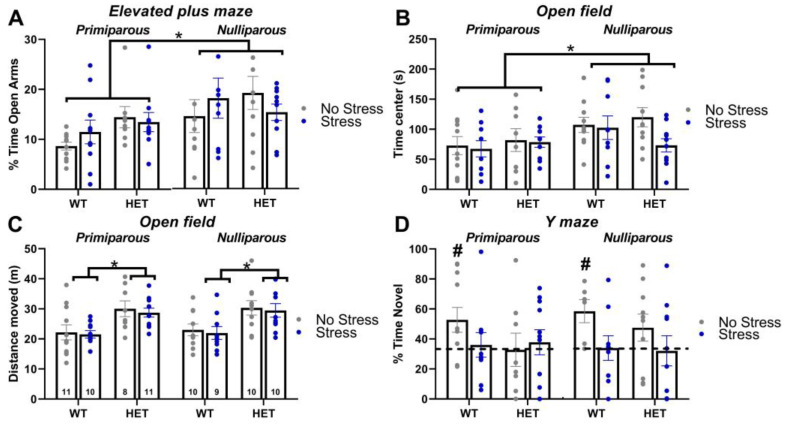
**Anxiety levels, exploratory behaviors and spontaneous alternation.** Anxiety levels were investigated in the elevated plus maze test (**A**) and the open field test (**B**), where locomotion was also assessed (**C**). Spontaneous alternation was evaluated in the Y-maze (**D**). * *p* < 0.05; # *p* < 0.05 versus chance level (indicated by the dotted line). N for each group is indicated in C. Data are expressed as mean ± SEM. Additional measures of locomotion in these tests are illustrated in Supplementary Figure S1.

Maternal experience increased the anxiety levels of all mice, independently of their genotype and stress condition (Supplementary Table S1). Primiparous females spent less percent time in the open arms of the maze than nulliparous mice [maternal experience effect: F(1,70) = 6.82, *p* = 0.01; Figure 2A]. Independently of maternal experience and stress exposure, HET mice were more active than their WT littermates [genotype effect: F(1,70) = 9.27, *p* = 0.01; Supplementary Figure S1A; Supplementary Table S1].

### 2.3. Open Field

As observed in the elevated plus maze, maternal experience increased the anxiety levels of all mice, independently of their genotype and stress condition (Supplementary Table S1). Primiparous females spent less time in the central area of the open field than nulliparous mice [maternal experience effect: F(1,70) = 6.16, *p* = 0.02; Figure 2B]. Mutant (HET) mice were more active than their WT littermates in the open field, independently of their stress and maternal conditions [Genotype effect on the distance traveled: F(1,71) = 24.81, *p* < 0.0001; Figure 2C; Supplementary Table S1] and they also moved faster [Genotype effect on the mean speed: F(1,71) = 24.57, *p* < 0.0001; Supplementary Figure S1B and Supplementary Table S1].

### 2.4. Y Maze

Only WT no-stress females (both nulliparous and primiparous) showed a significant preference for the novel arm (*t*-test versus the chance level in WT no-stress nulliparous and WT no-stress primiparous: t(5) = 3.26 and t(10) = 2.35, *p* = 0.02 and 0.04; Figure 2D), while all other groups displayed levels of spontaneous alternation that were indistinguishable from chance. Hence, both the mutant HET genotype and stress exposure induced a cognitive deficit in the Y-maze test. No difference among experimental groups was detected in locomotor activity during the test trial, as shown by the total distance moved and the mean speed (all main effects and their interactions, ns; Supplementary Figure S1C,D; Supplementary Table S1).

### 2.5. Social Interaction and Ultrasonic Communication

**Figure 3:**
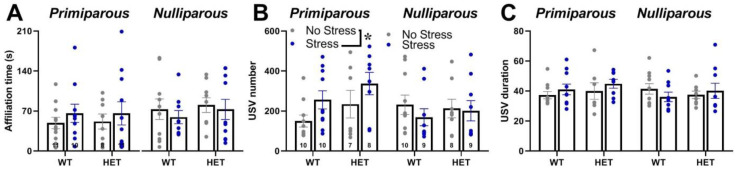
**Quantitative analysis of the social interaction and ultrasonic vocalizations (USVs) emitted during the social interaction test.** Social interaction was measured during a 3 min encounter with an adult NMRI WT female, computing the time spent in affiliative behaviors towards the stimulus (**A**). USVs were recorded during the direct social interaction test. Female experimental subjects were single housed in the testing cage for 72 h prior to testing. An unfamiliar adult stimulus female mouse was then introduced and left there for 3 min. Previous studies have shown that in these experimental settings, USVs are emitted only by the female intruder [54,55]. The number (**B**) and mean duration (**C**) of USVs were automatically measured using the software Sonotrack. Data are expressed as mean ± SEM. * *p* < 0.05.

**Figure 4:**
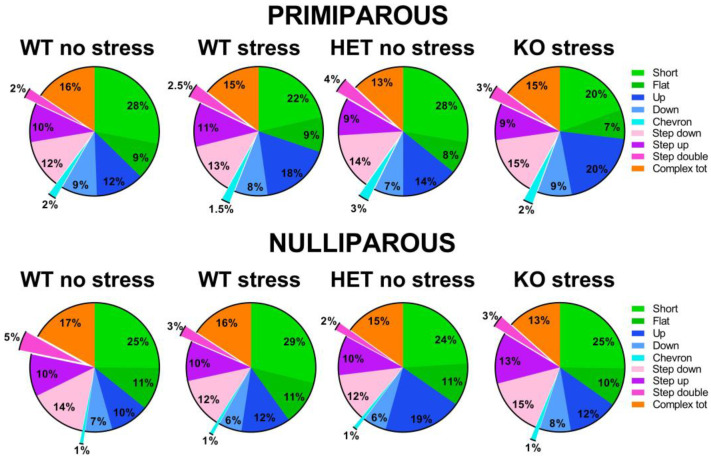
**Qualitative analysis of the ultrasonic vocalizations (USVs) emitted during the social interaction test.** Ultrasonic calls were automatically categorized as described before [22,56,57,58]. The “complex tot” category included all complex calls with more than 3 components. Data are expressed as mean percentages over the total number of USVs for each group.

Mutant genotype, exposure to stress or maternal experience did not affect the time spent in affiliative behaviors with an adult virgin female [all effects and interaction, ns; Figure 3A and Supplementary Table S1]. No difference among experimental groups was found in non-social behaviors. HET females also did not display quantitative differences in ultrasonic communication from their WT littermates, either in the USV number or mean duration [main effects of genotype, ns; Figure 3B,C; Supplementary Table S1]. Instead, stress increased the number of USVs, but only in primiparous females [stress × maternal experience interaction: F(1,63) = 4.29, *p* = 0.04; Figure 3B]. The qualitative analysis of call types revealed a highly comparable distribution of call types in all groups [all main effects and their interactions, ns; Figure 4 and Supplementary Table S1].

### 2.6. Corticosterone Blood Levels and Adrenal Gland Weight

**Figure 5:**
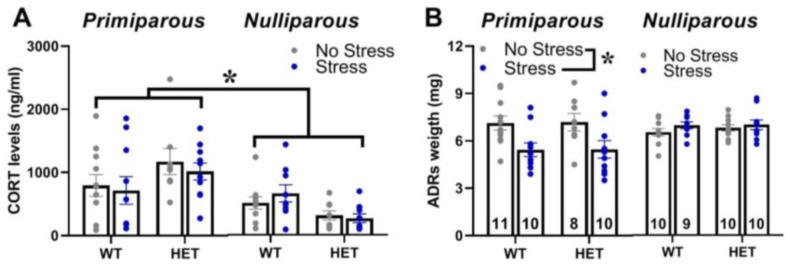
**Basal corticosterone blood levels and adrenal gland weight.** Basal corticosterone levels (**A**) and weight of both (adrenal glands (**B**)) were assessed one week after the end of behavioral testing, i.e., when mice were euthanatized (see also Figure in Section 4). * *p* < 0.05; N = 8–11 as indicated in (**B**). Data are expressed as mean ± SEM.

Maternal experience increased the basal blood levels of corticosterone for all females and this effect was observed in mice of both genotypes and stress conditions [main effect of maternal experience: F(1,69) = 13.01, *p* < 0.001; Figure 5A]. Maternal experience also promoted the effects of stress on adrenal gland weight, since stress reduced it only in primiparous females of both genotypes [interaction maternal experience × stress: F(1,70) = 13.25, *p* < 0.001; Figure 5B].

## 3. Discussion

In this study, we investigated the relative impact of mild chronic stress and maternal experience on the behavioral and endocrine profile of *Fmr1*-HET females. Our overall hypothesis was that stress could exacerbate the severity of the FXS-like phenotypes of *Fmr1*-HET mice and these effects would be exaggerated by maternal experience, i.e., when stress was applied during pregnancy. Hence, we hypothesized that the *Fmr1* mutation would enhance the vulnerability of female mice to chronic mild stress. Our findings demonstrated instead that the behavioral phenotypes of mutant females, i.e., hyperactivity and cognitive deficits, were unaffected by stress exposure even when applied during pregnancy. Furthermore, the *Fmr1*-HET females were slightly less sensitive to the effects of stress than their WT littermates, since the effects of stress on maternal behavior and spatial memory in the Y-maze tended to be more marked in WT mice. Our results also showed that certain effects of stress were promoted by maternal experience, as we expected, such as those detected on ultrasonic communication and adrenal gland -weight, but others were instead independent, i.e., the cognitive effects in the Y-maze. Furthermore, we demonstrated that maternal experience alone could enhance anxiety and basal corticosterone levels and these effects were equally observed in mice of both genotypes. 

Concerning the altered phenotypes of the *Fmr1*-HET females, our results here demonstrated mutant hyperactivity in the open field (Figure 2C) and cognitive deficits in the Y-maze (Figure 2D), without alterations in emotionality (Figure 2A,B), social interaction (Figure 3A) and ultrasonic communication (Figure 3B,C and Figure 4). This behavioral phenotype was mostly in agreement with previous reports, describing robust motor and cognitive alterations in mutant females, with inconsistent abnormalities in anxiety-like and social behaviors (e.g., [21,22,23]). Nonetheless, we previously described enhanced USV mean duration in HET females [21,22], associated with the emission of more complex ultrasonic calls compared to their WT littermates [22]. It is possible that the lack of USV qualitative alterations we found here in HET females could be related to the particular social housing conditions we used (e.g., housing first with a male, followed by a single housing period before being housed in same-sex pairs, see also Figure in Section 4) that were different from the standard social housing conditions used in our previous studies on *Fmr1*-HET mice [21,22]. Altogether, our results on the behavioral profile of *Fmr1* mutant females confirmed that motor and cognitive alterations represent the most robust FXS-like phenotypes, as it has been suggested for the *Fmr1*-KO males [23,59]. Furthermore, our findings add to the existing knowledge of the behavioral phenotypes of the *Fmr1*-heterozygous providing evidence of unaltered maternal care in *Fmr1*-HET females. Interestingly, the *Fmr1*-HET mice were undistinguishable from their WT littermates in their levels of nursing postures as well as pups’ care (Figure 1). This result is highly relevant to research on the *Fmr1* mouse line since previous studies have demonstrated that certain behavioral phenotypes of *Fmr1* males were associated with the genotype of their mothers [60,61,62], suggesting a role of alterations in maternal care, milk composition or immunological factors [62]. Our findings thus allow ruling out differences in maternal behaviors as the source of the effects of maternal HET mutation on the offspring behavioral phenotypes in the *Fmr1* mouse model.

In contrast to our hypothesis of a higher vulnerability of the *Fmr1* mutant genotype, the FXS-like behavioral phenotypes of the HET females, i.e., hyperactivity and cognitive deficits, were unaltered by stress, independently of maternal experience. Interestingly, while stress did not affect locomotor activity in all animals, it impaired the Y-maze performance in the WT mice without exacerbating the cognitive deficit of the HET females (Figure 2D). Hence, in disagreement with our initial expectations, the WT mice seemed more sensitive to the effects of stress, independently of maternal experience. This impression was further supported by the effects of stress on maternal behaviors (Figure 1), which were slightly more marked in the WT dams, where it reduced not only blanket posture and pup grooming as in the HET mice but also arched-back posture. Although in contrast with our initial hypothesis, these effects of stress on Y-maze performance and maternal behavior were in line with previous findings from WT rodents, highlighting stress-induced deficits in spatial memory [63,64,65] and maternal care [45,46,47]. Furthermore, a reduced behavioral and endocrine sensitivity of *Fmr1* mutant mice to chronic stress has been already suggested by previous studies (though they mostly focused on males) [22,37,38,57], and it could be interpreted as a deficit in the adaptive response to stressors [38]. This hypothesis, suggested also by previous authors [38], is supported by evidence showing that chronic stress-induced changes in dendritic branching and morphology in brain areas crucial for behavioral control, i.e., the amygdala in WT but not in *Fmr1*-KO male mice [38]. Interestingly, this attenuated sensitivity to stress displayed by the *Fmr1* mutants was limited to the behavioral response since no difference in corticosterone levels or adrenal gland weight was detected here in the HET mice (Figure 5). Our results are in agreement with previous studies from *Fmr1* mutant males, showing no difference in markers of the HPA axis functioning under basal conditions [38,66,67].

Overall, stress did not seem to be a highly powerful behavioral and endocrine modulator, at least when applied independently of maternal experience. Indeed, stress did not affect emotionality, locomotion, social interaction or any qualitative characteristic of ultrasonic communication in either the WT or HET mice. When combined with maternal experience, stress increased the number of USVs and reduced the adrenal gland weight. The limited behavioral effects of stress may be surprising considering the accumulating evidence from rodents describing several stress-induced effects on emotionality, locomotion and social behaviors [reviewed in [68,69]]. The lack of effects of stress on basal corticosterone levels is instead in line with a large body of data reviewed in [70,71], although discrepancies across studies exist, especially due to methodological differences [38,72]. Nonetheless, it should be noticed here that the long-term behavioral effects of stress were mostly investigated, i.e., between 5 and 7 weeks after the end of stress exposure (see Figure in Section 4), while previous studies were rather focused on short-term effects. 

Compared to stress, maternal experience was more efficacious in inducing behavioral and endocrine effects in mice of both genotypes. Primiparous WT and mutant females showed increased anxiety levels in both the elevated plus maze and open field tests than nulliparous mice together with enhanced basal corticosterone levels. These two effects are likely to be linked, as suggested by previous literature on corticosterone and emotionality [73,74,75,76]. The higher corticosterone levels of the primiparous females could also explain the reduced weight of adrenal glands that we detected when the maternal experience was combined with stress, possibly linked to a negative feedback mechanism on the HPA axis. Overall, the effects of maternal experience we detected in the present study were mostly “detrimental”, inducing blunted emotionality and high chronic stress levels; this finding could seem surprising, considering the general “beneficial” effects of maternal experience previously described, especially on cognition, in rats [reviewed in [77]]. Although an anxiogenic effect of maternal experience has already been described [39,41], comparisons with previous studies on maternal experience are complicated by differences in the experimental design. Among these, the most relevant are the inclusion of primiparous versus multiparous comparisons, differences in the interval from birth/weaning of the pups and mothers’ testing as well as the choice of control groups without a vasectomized male (that was instead employed in our study).

In conclusion, our findings suggest the presence of negligible interactions between environmental factors, such as stress and maternal experience, and the mutant genotype of *Fmr1* heterozygous female mice. Our results highlight instead the behavioral and endocrine impact of maternal experience in mice of both genotypes and their ability to interact with chronic mild stress. The present findings thus contribute to advancing our understanding of the complex environmental modulation of the pathological phenotypes of the *Fmr1* preclinical model of FXS. In terms of their translational validity for FXS patients, our results demonstrate the behavioral impact of environmental factors, such as stress and maternal experience, in FXS female heterozygous carriers. These preclinical findings, therefore, suggest that exposure to environmental adversity, especially during pregnancy, should be carefully controlled—and possibly avoided—in FXS female patients.

## 4. Materials and Methods

### 4.1. Breeding Procedures

**Figure 6:**
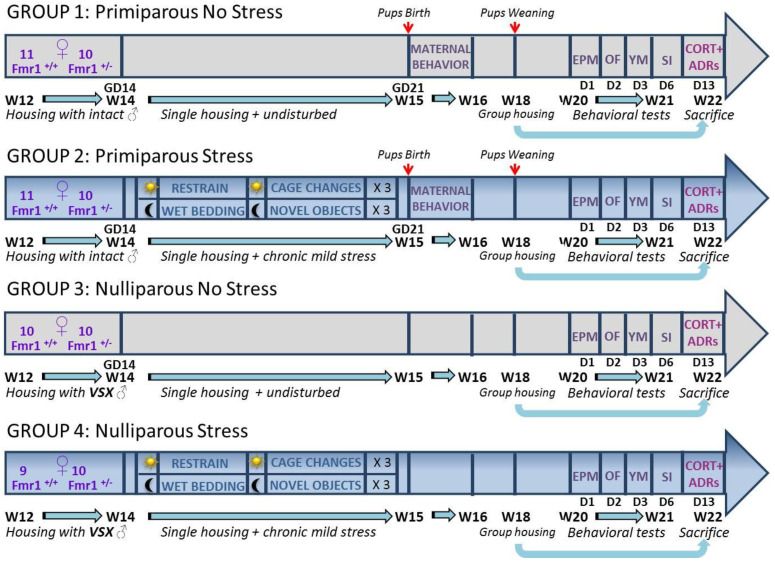
**Schematic representation of the experimental design of the study.** Unpredictable mild stress (groups 2 and 4) consisted of a 2 day-sequence that was repeated 3 consecutive times. On day 1, 3 sessions of 30 min restrain stress during the light phase were followed by overnight housing with wet bedding; on day 2, 3 sessions of sawdust and cage changes during the light phase were followed by overnight housing with novel objects. Control no-stress mice were instead left undisturbed. Group housing consisted of same-sex pairs of the same experimental conditions. W = week, referring to the age of the subjects; VSX = vasectomized; GD = gestational day; D = day; EPM = elevated plus maze; OF = open field; YM = Y-maze; SI = social interaction; CORT = corticosterone; ADRs = adrenal glands.

A total of 81 female mice (12 ± 1 week old) were used for the study: 42 “primiparous” [22 WT and 20 HET (+/−), n = 11/10 for each stress condition] and 39 “nulliparous” [19 WT and 20 HET (+/−), n = 9/10 for each stress condition]. C57BL/6J*Fmr1*^tm1Cgr/Nwu^ (B6) female mice were originally obtained from Neuromice.org (Northwestern University, IL, USA) and maintained on the C57BL6/J background for more than 10 generations. *Fmr1* heterozygous (+/−) females and their WT littermates used for this study were bred and genotyped in our animal facility at Bordeaux University as described in detail elsewhere [78]. They underwent the experimental procedures that are schematically summarized in Figure 6. 

Twenty-one C57BL/6J adult wild-type intact males [16 weeks old; purchased from Janvier (Le Genest St Isle, France)] were used for the «primiparous» experimental group, while twenty C57BL/6J adult wild-type vasectomized males [16 weeks-old; purchased from Janvier (Le Genest St Isle, France)] were employed for the «nulliparous» group. Breeding trios (2 females, either WT or HET, and 1 WT male, either intact or vasectomized for the primiparous or the nulliparous condition, respectively) were formed. Pregnancy was determined by the presence of semen in vaginal smears and its detection was identified on the first day of gestation (GD0). For both primiparous and nulliparous groups, the male was removed after two weeks from the formation of breeding trios (Figure 6). After removal of the male, pregnant and non-pregnant females were randomly assigned to either the no-stress or stressed group and they were individually housed in polycarbonate standard cages (37 × 21 × 15 cm in size; Tecniplast, Limonest, France), provided with sawdust bedding (SAFE, Augy, France) and a stainless steel wired lid. At 18 weeks of age, all females were housed in pairs with a female from the same experimental group and kept under these housing conditions until the end of the study. Food chow (SAFE, Augy, France) and water were provided ad libitum. All animals were maintained in a temperature- (22 °C) and humidity- (55%) controlled vivarium, under a 12:12 h light–dark cycle (lights on at 7 A.M.).

### 4.2. Stress Procedure

While females from the no-stress group were kept undisturbed in their home cage (Figure 6), mice from the stress group were exposed to the unpredictable stress procedure. This included the following 2-day sequence of events which was repeated three consecutive times:

- Day 1: 30 min of restrain stress (3 times each day during the light phase, with a 4 h interval) in perforated conical tubes (3 cm in diameter, 11.5 cm long; Becton Dickinson Labware Europe, France), followed by overnight housing with wet bedding (50 mL of water was added to floor sawdust of the home cage at the beginning of the dark phase).
- Day 2: multiple sawdust and cage changes (3 times each day during the light phase, with a 4 h interval), followed by overnight housing with novel objects (12 glass black beads, 1.5 cm in diameter were added in the home cage at the beginning of the dark phase).

Pregnant (primiparous group) and non-pregnant (nulliparous group) females were exposed to this sequence of events 3 times during one week (for pregnant females during the last week before parturition). This procedure was based on previous studies (e.g., [22,48,49,50,51]) and it is known to limit the habituation to stressful stimuli without using pain or nutritional manipulations. All pregnant females used for the study gave birth within 48 h after the last day of exposure to stress procedure. No alteration in the general health status of stressed animals emerged at the end of the stress paradigm. The health measures were taken by the animal caretakers through the daily observation of the animals in their home cages in order to assess both behavioral and physical indicators of welfare [79]. These included hunched posture, dull or sluggish movements, reduced locomotion/immobility, altered nest building and stereotypic behaviors, excessive grooming, absence of feces, rough hair coat, squinted eyes and skin abrasions/lesions [79].

### 4.3. Behavioral Testing Procedures

For the primiparous groups, maternal behavior was observed in their home cages twice a day for one hour (at 9.00 a.m. and at 5.00 p.m.) during the first six postnatal days of the pups, using an instantaneous sampling method (1 sampling/2 min, for a total of 30 sampling points/session). The following items were scored as absolute frequencies by an observer who was blind to the stress condition of the breeders [31,52,53]: (i) nursing postures, including arched-back nursing (the female is in an arched position over the nursing pups) and blanket nursing (the female is lying flat on top of the pups), (ii) non-nursing postures (the female is in contact with the pups, but not nursing, i.e., with no access to the nipples), (iii) licking/grooming of the pups.

For all subjects, behavioral tests began at 20 weeks of age (i.e., 2 weeks after weaning of the pups for the primiparous group) and were conducted as follows (see also Figure 6): on day 1, an elevated plus maze test for anxiety was administered, followed on day 2 by an open field test for locomotion and exploration, followed on day 3 by a spontaneous alternation test in a Y-maze and on day 6 by a direct social interaction test and the females’ estrous cycle assessment. All behavioral tests were carried out during the light phase of the cycle (between 9 A.M. and 4 P.M.) by an experimenter who was blind to the group assignment of the subjects. All mice were habituated to the experimental room for at least 30 min before the beginning of each behavioral test.

### 4.4. Elevated Plus Maze

The maze described in detail elsewhere [78,80] was placed 55 cm above floor level, in a quiet testing room with diffuse dim lighting (55 lux in the maze center). A digital camera was mounted above the maze, and images were transmitted to a PC running the Ethovision (Version 13, Noldus Technology, Wageningen, The Netherlands) tracking system. To begin a trial, the mouse was gently placed in the central square with its head facing one of the open arms and allowed to explore freely for 5 min. We measured the percent time in open arms as (time_(open arms)_/time_(open + closed arms)_) × 100.

### 4.5. Open Field

The open field consisted of a white opaque plastic arena (42 × 26 × 15 cm) under dim lighting conditions (55 lux). Each mouse was placed in the center of the arena and allowed to freely explore it for 10 min. Locomotor habituation, requiring longer testing sessions, was not assessed since it is known to be unaltered in *Fmr1*-KO adult mice (e.g., [78,81]; see also [23] for a review). Automated tracking of the videos obtained from a camera mounted above the open field was performed by Ethovision to analyze the distance traveled and average walking speed.

### 4.6. Y Maze

A grey plastic Y-maze (each arm measuring 8 × 42 × 15 cm, 120° spaced) was placed on a table 80 cm high, in a room presenting several extra-maze cues on its walls. For the habituation phase, one arm of the maze was closed; each mouse was introduced to the end of one maze arm and allowed to explore two arms for 5 min, while the access to the third arm was blocked by a transparent plastic door. After an interval of 3 min in a waiting cage, the testing phase began: the door of the blocked arm was removed and the mouse was allowed to explore all three arms for 2 min. Allocations of the start and blocked arms were counterbalanced within experimental groups. The entire maze was cleaned with a 70% ethanol solution between the habituation and test trials in order to avoid olfactory intra-maze cues. This Y-maze protocol has been previously used to test spatial memory in laboratory mice and it has been demonstrated to assess the performance in a hippocampal-dependent task based on the exclusive use of extra-maze spatial cues [82]. Time spent in each arm during the habituation and testing phases was scored by EthoVision through automatic tracking of the videos collected from a camera mounted above the maze center. We measured the percent alternation rate as (time_(novel arm)_/time_(all arms)_) × 100. In addition, the total distance and average speed were measured.

### 4.7. Social Interaction and Ultrasonic Communication

Stimulus mice used for the direct social interaction test were adult (12 weeks of age) female NMRI mice, as this strain is commonly employed in social studies [83,84], especially those using the *Fmr1*-KO mouse model [21,31,85]. This strain is often chosen since it is characterized by high levels of sociability and it facilitates the behavioral analysis during social encounters with B6 mutants because of its albino phenotype. NMRI mice were purchased at 10 weeks of age from Janvier (Le Genest-Saint-Isle, France), housed in groups of 3–4 per cage and left undisturbed for 2 weeks before being used in behavioral tests. The choice of the age of stimulus mice was based on previous studies with females in the resident-intruder setting [84,86,87], all using adult stimulus females. Indeed, in this experimental context, adult stimulus females do not emit ultrasonic vocalizations (USVs) which are instead mostly uttered by the resident female [86,87], as demonstrated by alternately anesthetizing each pair member. The absence of “double calls”, i.e., overlapping in their timing, but with different, non-harmonic, characteristics (e.g., different peak and mean frequency, modulation), was also confirmed here by the inspection of all spectrograms.

Female experimental subjects (WT or *Fmr1*-HET mice) were isolated in the testing cage for 72 h, in order to induce a status of resident in experimental females and, therefore, promote the emission of ultrasonic vocalizations (USVs) towards an adult female intruder [84]. An unfamiliar NMRI stimulus female mouse was then introduced into the testing cage of either male or female subjects and left there for 3 min.

Testing sessions were recorded by a camera placed on the side of the cage and the videos were analyzed with Observer XT (Noldus, Wageningen, The Netherlands). One observer who was unaware of the experimental conditions of the animals scored the behavior of the test mice, quantifying the time spent performing affiliative behaviors [21,31,85], i.e., sniffing the head and the snout of the partner, its anogenital region or any other part of the body; contact with the partner through traversing the partner’s body by crawling over/under from one side to the other or allogrooming. Non-social activities were also measured [31,85]: rearing (standing on the hind limbs sometimes with the forelimbs against the walls of the cage); digging; self-grooming (the animal licks and mouths its own fur).

An ultrasonic microphone UltraSoundGate Condenser Microphone CM 16 (Avisoft Bioacoustics, Berlin, Germany) was mounted 2 cm above the cover of the testing cage. Recordings were then analyzed through Sonotrack Software (version 1.4.7, Metris B.V., Hoofddorp, The Netherlands) This software fully automatically recognizes several different USV types and also calculates quantitative parameters including the total number and mean duration of the calls. Based on previous literature on call types [22,56,57,58,87,88,89], the following USV types were selected for automatic recognition in our dataset: short, flat, (ramp) up, (ramp) down, chevron, step-up, step-down, step-double (split), complex-3, complex-4, complex-5, complex-5+. Their characteristics are described in detail elsewhere [22,56,57,58]. Definitions of call types were mutually exclusive. Overlap of components was removed when more than 70% to prevent wrong call durations. Short gaps between components in both frequency (≤6 kHz) and time (≤5 ms) were interpolated (gaps can be caused by changes in microphone sensitivity or direction of vocalization). The calls classified as “complex-3 component” and “+3 component” were summed up into a “total complex” category, as in previous studies [22,56,57,58].

The estrus phase of female mice was assessed by analysis of vaginal smears [90] performed on the testing day in both the experimental subjects and NMRI stimulus mice. The evaluation of the *Fmr1* WT and HET (+/−) females used as experimental subjects was conducted after their testing in order to minimize the potential stress effects of the manipulation necessary for determining the estrous phase. Stimulus NMRI females and experimental subjects were all in the diestrus phase at the moment of social testing.

### 4.8. Assessment of Corticosterone Blood Levels

One week after the end of behavioral testing (Figure 6), mice were euthanized by cervical dislocation (all samples were obtained in the morning between 8 and 9 A.M.); both adrenal glands were immediately dissected and weighted. Blood samples were obtained from trunk blood, collected in heparinized tubes and stored on ice. After centrifugation at 3000 r.p.m. for 10 min, the supernatant was stored at −80 °C until the assay was performed. Corticosterone measures were assessed in duplicate by ELISA using a commercial kit (Cayman Chemical Company, Ann Arbor, MI, USA).

### 4.9. Statistical Analysis

The normality of data distribution was verified using the Shapiro–Wilk test. Except for data on maternal behavior (which were obviously obtained only from the primiparous group and, therefore, were analyzed without the variable of maternal experience), all data were analyzed through a 2 × 2 × 2 ANOVA with maternal experience, stress and genotype as the between-subject factors. Alternation rates from the Y-maze test were instead analyzed with t-tests from the chance level (corresponding to the value of 33.33%), as in previous studies including those involving *Fmr1* mutant mice (e.g., [22,57,91]). This type of analysis can be used as an alternative to the ANOVA of the percent time spent in the novel arm of the Y-maze and it allows for identifying a deficit in Y-maze learning, revealed by a performance undistinguishable from the chance level, also in the absence of significant group differences. Furthermore, the comparison with the chance level allows for verifying that the test has worked properly in the control mice, thus establishing a proper baseline for evaluating the effects of experimental conditions. Post hoc comparisons were performed using Tuckey’s test when a significant interaction was detected.

Analyses were conducted using the software StatView (SAS institute, 5.0.1, NC, USA) and SPSS (PAWS Statistics 18, Chicago, IL, USA) and α was set at 0.05. Results are expressed as mean ± SEM throughout the text. The exact number of mice is indicated in the legend of each figure; slight differences in the number of mice per experimental group may be due to technical reasons (e.g., problems in behavioral scoring or recording) or to the exclusion of outliers (using Grubbs’ ESD test adapted for small sample size).

## Data Availability

The datasets used and analyzed during the current study are available from the corresponding author upon reasonable request.

## Supplementary Materials

The supporting information can be downloaded at: https://www.mdpi.com/article/10.3390/ijms241411398/s1.
