# Supplementary material for: The Impact of Mild Chronic Stress and Maternal Experience in the Fmr1 Mouse Model of Fragile X Syndrome
Source: Int J Mol Sci. 2023 Jul 13;24(14):11398. doi: 10.3390/ijms241411398 (PMC10380347; doi:10.3390/ijms241411398)
Supplement: Supplementary file 1 [file ijms-24-11398-s001.zip › ijms-2451318-supplementary-Figure.pdf]

International Journal of
Molecular Sciences

MDPI

Article

The Impact of Mild Chronic Stress and Maternal Experience in the *Fmr1* Mouse Model of Fragile X Syndrome

Enejda Subashi †, Valerie Lemaire †, Valeria Petroni and Susanna Pietropaolo *

Univ. Bordeaux, CNRS, EPHE, INCIA, UMR 5287, F-33000 Bordeaux, France
* Correspondence: susanna.pietropaolo@u-bordeaux.fr
† These authors contributed equally to this work.

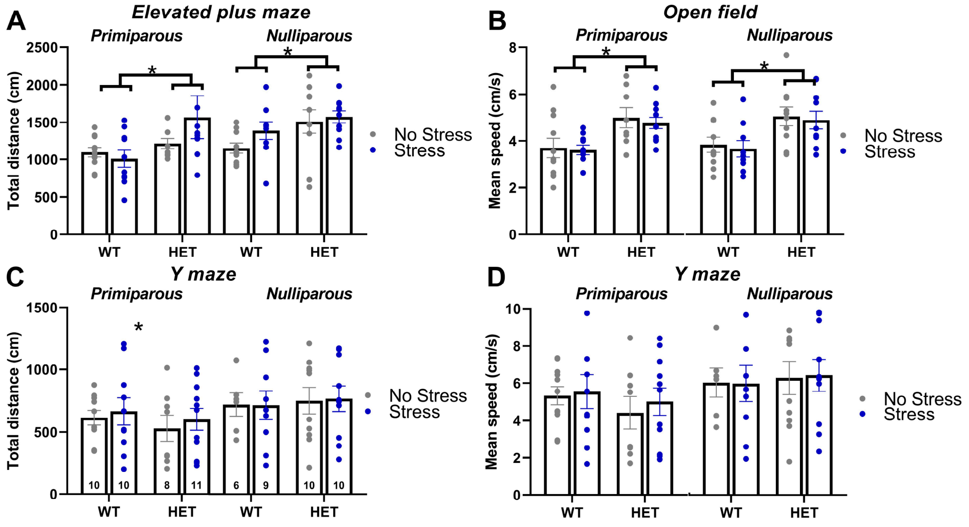

Figure S1: Additional behavioral measures in anxiety and memory tests. Locomotion and average speed were assessed in the elevated plus maze (A), open field (B), and during the test trial of the Y maze (C and D). Data are expressed as mean±SEM. * p<0.05. N is indicated in C.
