# Supplementary material for: The Impact of Mild Chronic Stress and Maternal Experience in the Fmr1 Mouse Model of Fragile X Syndrome
Source: Int J Mol Sci. 2023 Jul 13;24(14):11398. doi: 10.3390/ijms241411398 (PMC10380347; doi:10.3390/ijms241411398)
Supplement: Supplementary file 1 [file ijms-24-11398-s001.zip › ijms-2451318-supplementary-Table.pdf.pdf]

Table S1: Statistical outcome of the maternal experience x genotype x stress ANOVA of all behavioral variables

Test	Variable	Maternal exp			Maternal exp x Genotype			Maternal exp x Stress			Maternal exp x Stress x Genotype			Genotype			Stress			Genotype x Stress		
		DF	*F*	*p*	*DF*	*F*	*p*	*DF*	*F*	*p*	*DF*	*F*	*p*	*DF*	*F*	*p*	*DF*	*F*	*p*	*DF*	*F*	*p*
EPM	*% time open arm*	1-70	6.82	**0.01**	1-70	0.66	0.42	1-70	0.08	0.77	1-70	0.25	0.62	1-70	1.7	0.20	1-70	0.05	0.83	1-70	2.39	0.13
	total distance	1-70	3.39	0.07	1-70	0.11	0.75	1-70	0.01	0.93	1-70	2.38	0.13	1-70	9.27	**0.01**	1-70	2.01	0.16	1-70	0.45	0.51
OF	*time in center*	1-70	6.16	**0.02**	1-70	0.81	0.37	1-70	1.08	0.30	1-70	1.20	0.29	1-70	<0.01	0.93	1-70	2.13	0.15	1-70	0.95	0.33
	total distance	1-71	0.17	0.68	1-71	<0.01	0.98	1-71	<0.01	0.99	1-71	0.02	0.89	1-71	24.81	**<0.0001**	1-71	0.43	0.51	1-71	0.01	0.94
	mean speed	1-71	0.15	0.70	1-71	<0.01	0.98	1-71	<0.01	0.99	1-71	0.02	0.88	1-71	24.57	**<0.0001**	1-71	0.42	0.52	1-71	0.02	0.90
YM	*total distance*	1-67	3.39	0.07	1-67	0.82	0.37	1-67	0.09	0.76	1-67	<0.01	0.94	1-67	0.10	0.75	1-67	0.15	0.70	1-67	0.06	0.81
	mean speed	1-67	3.19	0.08	1-67	0.97	0.33	1-67	0.12	0.73	1-67	<0.01	0.97	1-67	0.16	0.69	1-67	0.19	0.67	1-67	0.04	0.84
SI	*time affiliation*	1-70	1.41	0.24	1-70	0.17	0.68	1-70	1.41	0.24	1-70	0.43	0.84	1-70	0.27	0.61	1-70	0.05	0.83	1-70	<0.01	0.93
USVs	*nb*	1-63	1.43	0.24	1-63	1.23	0.27	1-63	4.29	**0.04**	1-63	0.16	0.69	1-63	1.72	0.20	1-63	0.98	0.33	1-63	0.12	0.73
	dur	1-63	0.60	0.44	1-63	0.39	0.54	1-63	1.19	0.28	1-63	0.46	0.50	1-63	0.41	0.53	1-63	0.34	0.56	1-63	0.76	0.39
	% short	1-63	0.19	0.66	1-63	<0.01	0.99	1-63	3.81	0.06	1-63	<0.01	0.98	1-63	0.82	0.37	1-63	1.62	0.21	1-63	0.13	0.73
	% flat	1-63	3.43	0.07	1-63	0.09	0.77	1-63	0.08	0.78	1-63	0.17	0.68	1-63	1.41	0.24	1-63	0.39	0.54	1-63	0.13	0.72
	% up	1-63	1.03	0.31	1-63	0.08	0.78	1-63	3.47	0.07	1-63	1.08	0.30	1-63	2.73	0.10	1-63	0.78	0.38	1-63	0.97	0.33
	% down	1-63	3.20	0.08	1-63	0.52	0.48	1-63	0.25	0.62	1-63	0.03	0.86	1-63	0.17	0.68	1-63	0.07	0.80	1-63	1.18	0.28
	% chevron	1-63	3.21	0.08	1-63	0.62	0.43	1-63	2.21	0.14	1-63	0.45	0.51	1-63	0.24	0.62	1-63	1.15	0.29	1-63	2.14	0.15
	% step down	1-63	0.02	0.90	1-63	1.16	0.28	1-63	0.09	0.77	1-63	2.86	0.10	1-63	0.48	0.49	1-63	0.12	0.73	1-63	0.39	0.54
	% step up	1-63	0.32	0.57	1-63	2.33	0.13	1-63	0.32	0.58	1-63	0.98	0.33	1-63	0.21	0.65	1-63	0.44	0.51	1-63	1.17	0.28
	% step double	1-63	0.87	0.35	1-63	0.90	0.35	1-63	0.05	0.83	1-63	2.52	0.12	1-63	0.10	0.76	1-63	0.19	0.66	1-63	0.36	0.55
	% complex tot	1-63	1.47	0.23	1-63	<0.01	0.96	1-63	0.07	0.80	1-63	<0.01	0.94	1-63	0.75	0.39	1-63	<0.01	0.98	1-63	0.22	0.64

EPM= elevated plus maze. OF= open field. SI= social interaction. USVs= ultrasonic vocalizations. Nb= number. Dur= mean duration. Slight differences in the exact number of mice between tests or variables are due to technical reasons (e.g.. loss of behavioral video recordings. animals falling from the elevated plus maze) or to the exclusion of outliers (using Grubbs' ESD test adapted for small sample size). Bold characters indicate significant values (p<0.05).
